# Dispersion modeling and health risk assessment of VOCs emissions from municipal solid waste transfer station in Tehran, Iran

**DOI:** 10.1186/s40201-017-0268-0

**Published:** 2017-03-07

**Authors:** Maryam Sarkhosh, AliAkbar Shamsipour, Kamyar Yaghmaeian, Ramin Nabizadeh, Kazem Naddafi, Seyed Mohsen Mohseni

**Affiliations:** 1 Department of Environmental Health Engineering, School of Public Health, Tehran University of Medical Sciences, Tehran, Iran; 2 Department of Physical Geography, Faculty of Geography, University of Tehran, Tehran, Iran; 3 Center for Air Pollution Research (CAPR), Institute for Environmental Research (IER), Tehran University of Medical Sciences, Tehran, Iran; 4 Department of Environmental Health Engineering, School of Public Health, Shahid Beheshti University of Medical Sciences, Tehran, Iran

**Keywords:** TAPM, Waste transfer station, Health risk assessment, VOCs, air pollution

## Abstract

**Background:**

The waste transfer stations (WTSs) is one of the most important factors affecting on environment and human health. This research is aimed to evaluate health risk of VOCs among WTS personnel and provide a model for dispersion of VOCs. The Air Pollution Model (TAPM) is able to simulate WTS emissions dispersion over each town.

**Result:**

GC-MS was used to analysis collected gas samples to detect and estimate carcinogenic and non-carcinogenic VOCs health risks. The total lifetime cancer risk values for the all personnel (3.30E-05), was more than acceptable limit (1.00E-06). Furthermore, hazard ratio (HR) of 1,2,3-trimethylbenzene, 1,3-dichloropropane, toluene, m,p-xylene and ethylbenzene were 3.7, 1.9 E-01, 4.4 E-03, 5.5 E-02 and 1.5 E-03, respectively, and total HR of the mentioned compounds were more than accepted limit (HR < 1.00). IOA is 0.85 and RMSE is 2.16 and TAPM has a good performance. The VOCs level is considerable in 1600 m far from the WTS particularly in summer that require more attention.

**Conclusion:**

The exposure to VOCs was at a high level in WTS, and some controlling strategy should be used for decreasing the pollution and protecting the citizens and personnel against non-cancerous and cancerous risks.

## Background

Volatile Organic Compounds (VOCs) have considerable roles as the secondary precursors of air pollutants and bad influences on human health [1, 2]. Several VOCs such as alkanes, alkenes, aromatic hydrocarbons, oxygen and nitrogen including materials as well as terpenes can contribute to produce the tropospheric ozone and secondary organic aerosols (SOAs), which are the key components to increase PM_2_._5_ pollution [3]. However, several VOCs categorized as class 1 carcinogens, including benzene, formaldehyde and acetaldehyde, have resulted in cancer. In addition, exposure to other VOCs including toluene, ethylbenzene and trichloroethylene, may have bad impacts on humans health [4, 5]. During past recent decades, broad studies there have been conducted on the VOC levels and scenarios such as atmospheric and industrial environment as well as vehicle sources [1, 6–8], but only a few studies are available about reactivity contributions of VOC spreaded from waste transfer stations (WTSs). Transfer station is a facility which is used to store waste temporarily in regions near to urban areas [9]. WTSs have bad influences on neighborhoods and personnel like noise and emissions of bad odors relating to solid waste and oil of the transfer vehicles [9]. The aim of this research is to assess VOCs emission from WTSs in Tehran and to depict VOCs dispersion modeling based on relative softwares. In addition, the current research has three specific goals. The first one is to present quantitative information on the VOCs levels in WTS as there are very limited research in a WTS site ambient air with no information about the dispersion of VOCs. The second goal is to present information of chronic health impacts on WTS personnel including non-cancer or cancer risk of VOCs, which could be induced through breathing in such sites because there are limited information about evaluation of the health risk caused by emitting VOCs from WTS and most earlier studies emphasized on evaluation of their health risk caused by landfills [3, 10]. Furthermore, there is no extensively accepted standard process for assessing risk of gases produced from landfills and WTS. The third goal is to develop the knowledge about distribution of emitting VOCs from WTS. Most of earlier studies emphasized on the dispersing VOCs in landfill or waste treatment plants [3, 11], but not in the WTS.

## Methods

### Site description

The capital of Iran, Tehran, is a large city located in a relatively restricted region on the southern foothills of the Alborz Mountains UTM (35° 34–35° 50′ N and 51° 08–51° 37′E). The city covers a total area of 730 km^2^ and has a population of 9 million residents. Most of its residents experience daily air with a poor quality [1].

**Fig. 1:**
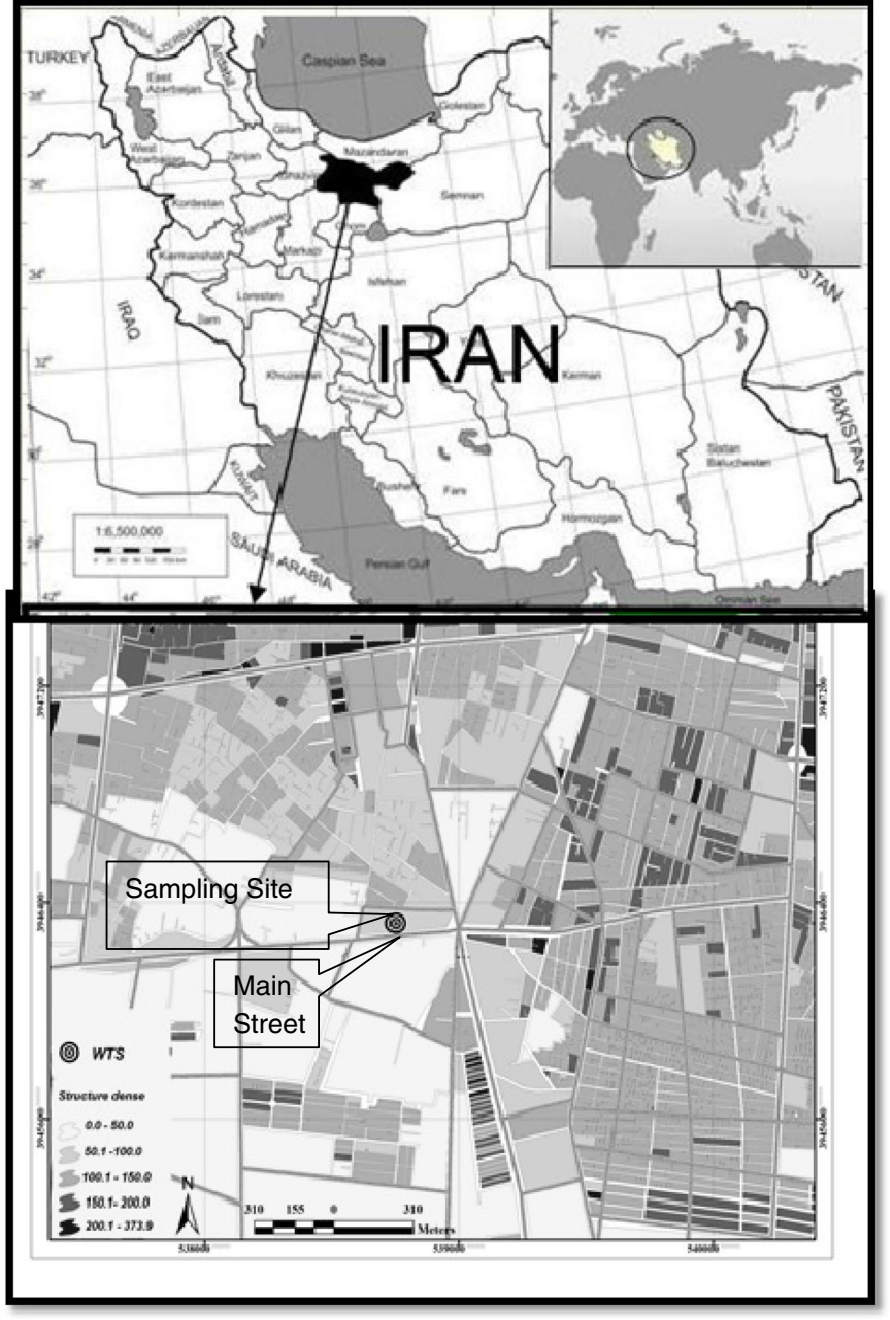
Geographical location of Tehran and the measurement site

The study was a full-scale study on WTSs in Tehran during the winter and summer 2015 (Fig. 1).

Tehran is producing the large amount of waste per day which is estimated to be 7.7 million ton in average and yearly generated waste in Tehran has had an increasing trend [12]. The MSW includes customers, transfer stations and landfills. The transfer stations accounts for compacting the wastes collected from customers and loading them into semi-trailers to transfer to final disposal [13]. The selected WTS with capacity of 300–350 t/day loads which commonly consist of household wastes, local restaurants leftover, waste papers and consumed plastics.

### Air sampling and analysis

Air was sampled at the dumping site on selected days (EPA Sampling Schedule 2015), representing the two different seasons of the year.

The air sampling was performed by adsorbing air on tubes packed with Tenax-TA and Carbopack-B by means of pumps with constant flow (SKC). A flow meter was used to daily control the pump flow rate at about 100 ml min^_1^. The sampling period lasted almost 2 h. All sampling adsorbent tubes were brought to the laboratory for analysis, within 8 h. The products trapped on the activated carbon are extracted with CS_2_. The analysis was performed by a gas chromatography (GC, Agilent 7890N, Agilent Co.) equipped with mass spectrometry (MS, Agilent 5975C, Agilent Co.). At first, the preconcentration of achieved gas samples were performed using cryogenic liquid nitrogen, based on the procedure of EPA methods TO-14 and TO-15. The system applied an Agilent Innowax, 30 m × 0.25 μm × 0.5 μm capillary column (HP5-MS, 0.25 mm film thickness). The constant flow of 1.4 mL min^_1^ was applied to run the column. The injection volume was 1 μL in the splitless mode at 200 °C, and the applied carrier gas was Helium with a purity of 99.99% at constant flow rate of 1 ml min^−1^. The temperature program applied for the column was as follows: initial temperature of 40 °C, enhanced at a rate of 4 °C/min to 70 °C, then kept for 4 min, raised to 200 °C at 10 °C/min, and held for 3 min. The internal standard approach was applied for quantitative analysis of all the recognized VOCs emissions, and the outcomes were stated in μg.m^−3^. Limits of detection (LOD) were below 0.1 μg.m^−3^.

### Health risk assessment

Health risk has been evaluated for protecting public health against difficulties of air pollution focusing on the VOCs chronic impacts rather than acute toxicity [8, 14]. The effects of human health can be classified as two groups of noncancer or cancer risks. The approximation of cancer and non-cancer risks were evaluated for VOCs exposure over inhalation based on the US Environmental Protection Agency (USEPA) standard procedure. The non-cancer risk was measured with dividing the daily level of each compound (C in μg m^−3^) by its corresponded Reference Concentration (RfC in μg.m^−3^), considered as hazard ratio (HR):

1 $$ \mathrm{H}\mathrm{R}=\frac{\mathrm{C}}{\mathrm{R} F\mathrm{C}} $$

The reference levels for all VOCs were extracted from the reference [15]. The lifetime cancer risk (LCR) was defined as multiplying predicted value of unit risk (UR) of each compound by the mean daily levels (in μg.m^− 3^) of every compound, as demonstrated in Eq. (2) [16].

2 $$ \mathrm{L}\mathrm{C}\mathrm{R}=\sum \mathrm{C}\times \mathrm{U}\mathrm{R} $$

### The air pollution model

In current research, TAPM (The Air Pollution Model) V4 and SURFER® 10 (Golden Software, Inc.) were applied to describe dispersion level maps. The output data obtaining from TAPM were entered in Surfer® 10. TAPM was improved by Australian Commonwealth Scientific and Research Organization (CSIRO) [17, 18]. The resulted pollution confirms well performance of TAPM for reactive pollutants in regulativeuses of earlier confirmation research as an urban airshed modeling tool. The TAPM applies Eulerian Grid Module (EGM), Lagrangian Particle Module (LPM), the Plume Rise Module and the Building Wake Module for predicting dispersion according to chemistry, fluid dynamics, meteorology, terrain and land use [17]. Pollution information are run in three modes of tracer, chemistry and dust. In current study, passive tracer mode was used to run pollution data [19]. The simulation was performed through four networks that each of them containing 25 × 25 cells. The meteorological input external network covered 35 at 35 km. Twenty-five levels were considered in the vertical direction. Grid points of each network for pollution contained 36 × 36 cells and the interval of these points were 10 km, 5 km, 2 km and 700 m. In this study, TVOC has selected as tracer in the chosen days for summer and winter. Root Mean Square Error (RMSE) and Index of Agreement (IOA) were applied to evaluate the performance of TAPM [20].

## Results and discussion

### Levels of VOCs in WTS

In this study, a total of 14 dominant VOCs were quantified during the two seasons. Only prevalent VOC emissions with an average level more than 20% of the total level of the several families were applied for decreasing analysis uncertainty. The outcomes (Table 1) indicate that limonene, acetaldehyde and propylene are the major organic compounds released by MSW, as also seen in similar research [11, 21]. Other significant compounds were toluene, 1,2,3trimethylbenzene, xylene, dichloropropane and ethylbenzenethat were detected. Among the aromatics compounds, toluene was detected at the highest concentrations. Toluene concentrations ranged from 9.54 to 36.21 μg.m^−3^ (Mean ± SD = 21.62 ± 8.11 μg.m^−3^) in summer and 3.01 to 42.15 μg.m^−3^ (Mean ± SD = 13.92 ± 1.17 μg.m^−3^) in winter.

**Table 1:** Summary of total concentrations (μg.m^−3^)

VOCs	winter			summer		
	Mean	Minimum	Maximum	Mean	Minimum	Maximum
Propylene	80.86	52.70	152.02	60.60	20.16	90.08
1,2,3Trimethylbenzene	12.06	0.02	87.20	17.87	7.28	26.04
Acetaldehyde	92.42	35.57	225.20	85.88	30.65	123.74
limonene	171.26	62.85	350.10	123.32	41.2	183.45
1,3-Dichloropropane	2.45	0.01	10.02	5.50	2.17	9.53
Toluene	13.92	3.01	42.15	21.62	9.54	36.21
m,p-Xylene	18.03	0.91	45.2	11.05	4.69	15.68
Ethylbenzene	2.32	0.01	9.02	2.10	0.85	4.30

During sampling period, limonene was found as the most abundant species (from 41.2 to 183.45 μg.m^−3^ in summer and increased to 350.10 μg.m^−3^ in winter). Moreover, it is clear that terpenes are the main component emitted from MSW, especially in fresh waste emissions [11]. These components can also emit from the waste of vegetable and the organic material first degradation [10, 11]. In addition to, terpenes can be responsible for increasing PM_2.5_ due to contribute to the formation of tropospheric ozone and secondary organic aerosols (SOAs) [6, 22].

Acetaldehyde was presented at 92.42 μg.m^−3^, in winter and 85.88 μg.m^−3^ in summer, followed by the alkene, aromatics and chlorinated compounds, including propylene, toluene, xylene, ethylbenzene, trimethylbenzene, and dichloropropane. In this study, the recognized alkanes were more than earlier report for WTS [23]. This implied that the high content of organic matters, including the content of vegetable wastes or meat, whose consumption generally increase during summer, contributed to the high trace component emission.

### Health risk assessments

The possible chronic health effects (cancer and non-cancer risk) of exposure to VOCs by inhalation were evaluated among personnel of the station. In current research, for all quantified VOCs, 5 and 2 VOCs were only evaluated non-cancer and cancer risk, respectively, because of the lack of reference levels [15]. If the HR is more than or equal to 1.0, the risk indicates non-carcinogenic impacts of the chemicals [24]. The mean HR of 1,2,3trimethylbenzene, 1,3-dichloropropane, toluene, m,p-xylene and ethylbenzene were 3.7, 1.9 E-01, 4.4 E-03, 5.5 E-02 and 1.5 E-03, respectively. 1,2,3trimethylbenzene was only quantified among mentioned VOCs in more than 70% of the samples. The estimated individual mean of non-carcinogenic risks (i.e., HRs) for 1,2,3trimethylbenzene are not insignificant at the site of WTS as the HRs are higher than one. In the case of exposure to two or more dangerous compounds, the combination of their effects have to be considered rather than the independent effects of each of them. Though the individual substances may aim various humans organs, total cumulative lifetime non-carcinogenic risks should be considered as additive in the lack of contrary data [24]. In current research, 3.95 was obtained for HR of all compounds. Thus, exposure to VOCs can influence on the health of residents and personnel. It should be noted that 1,2,3-trimethylbenzene is not involved in the USEPA Hazardous Air Pollutants list (USEPA, 1994), which require more attention in next research according to current study.

“definite risk”, “probable risk”, “possible risk” and “negligible risk” was defined for compounds with LCRs more than 1 E-04, between 1E-04 and 1E-05, between 1E-05 and 1E-06, and less than 1E-06, respectively [16, 24]. In current study, mean LCRs of 1,3-dichloropropene and ethylbenzene were 2.31E-05 and 9.93E-06 respectively for worker exposures to air. 3.30E-05 was obtained for total cancer risk of all personnel. It is mentioned that the personnel have been posed possible risk cancer risks at WTS.

In general, 1,2,3trimethylbenzene as non-cancer risk mainly contributes to the chronic health effects in WTS; while 1,3-dichloropropene and ethylbenzene mainly contribute to risks of cancer as Group 2B. Therefore, health risks evaluation showed that the personnel might be suffered from both acute and chronic health risks at WTS. Therefore, several controlling methods should be performed for decreasing the pollution and protecting the residents and personnel against the risks of cancer and non-cancer.

### Dispersion modeling

**Fig. 2:**
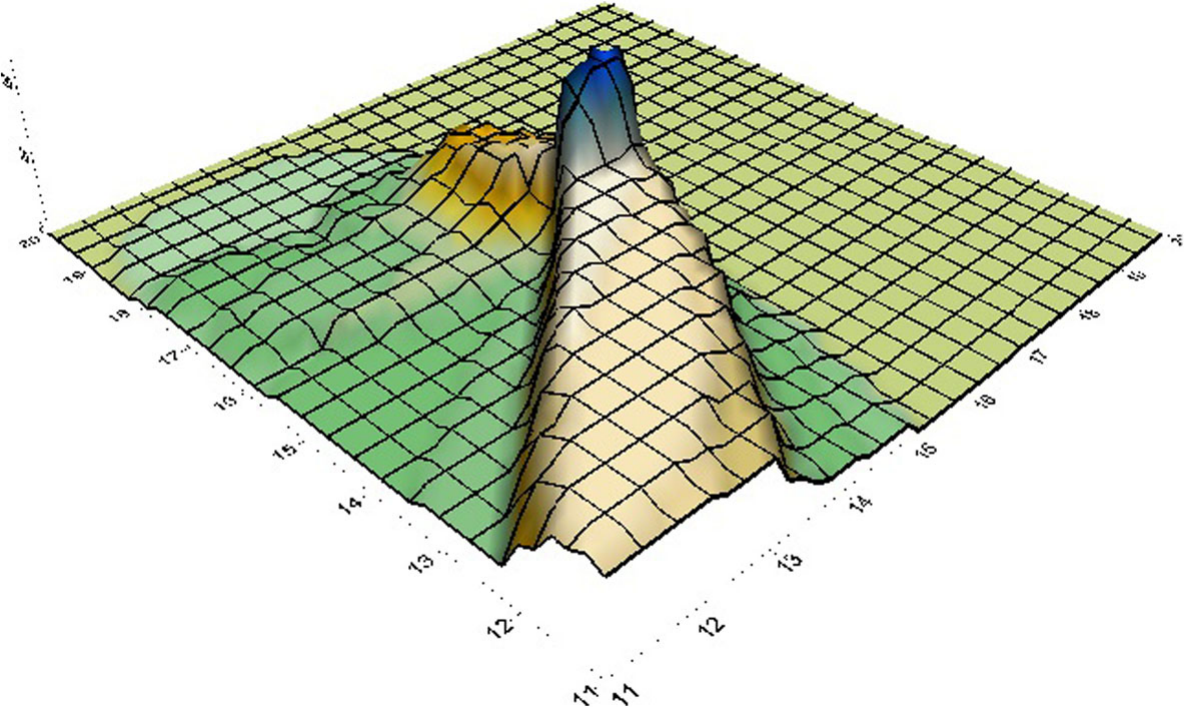
3D surface map for TVOC in July 10, 2015

**Fig. 3:**
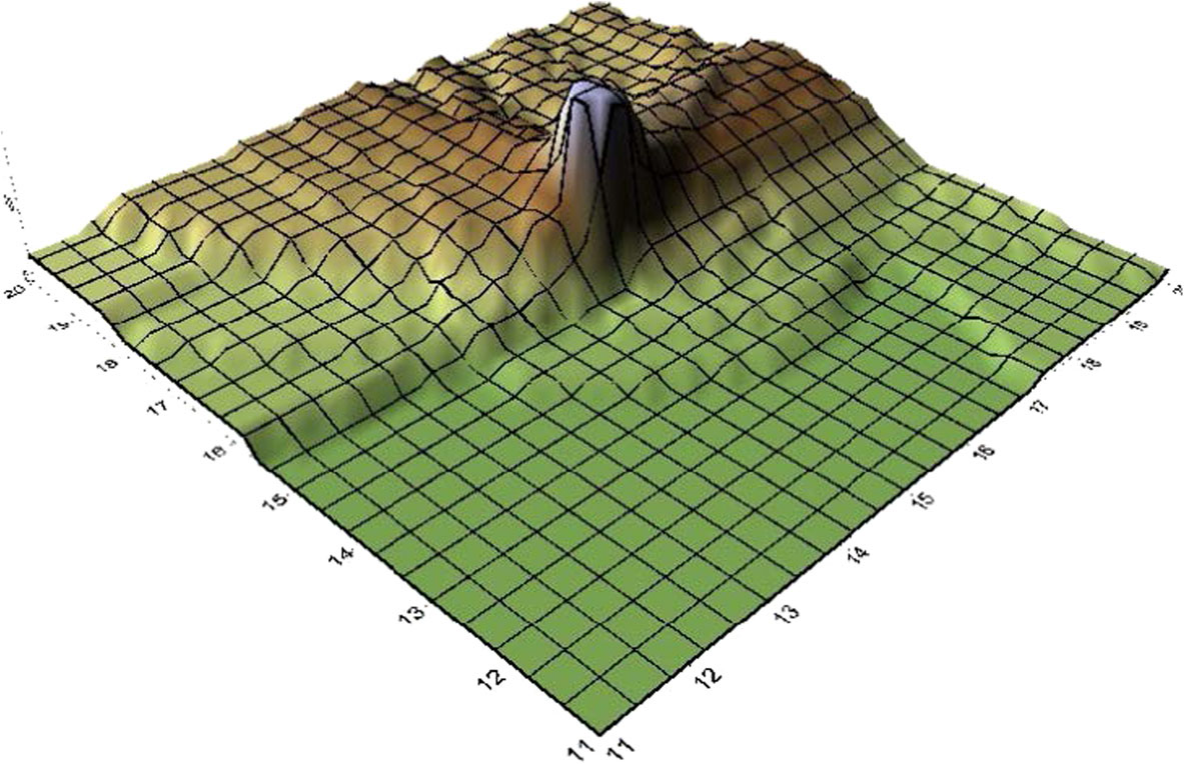
3D surface map for TVOC in March 1, 2015

Important indices of the diffusive feature of a site are parameters of atmospheric dilution [19]. They are helpful to estimate the yearly mean level distribution of gaseous wastes released from any facility. In current research, the used meteorological information were created using TAPM model at 700 m altitude in 2015. Direction of wind is one of the main parameters for determination of pollutant dispersion [25]. TVOC level hypsometric mapping at altitude of 10 m shows the southwest and northeast winds direction dominated in the WTS area over all sampling period. The pattern for dispersion of TVOC are so similar to Fig. 2 in summer for other days as dominated direction of winds is from SW of WTS in which there are few residents and buildings, while dense population and buildings can be observed in North. Figure 3 showed that patterns of TVOC dispersion for 1th March are the dominant winds from the NE. Therefore, more people will be exposed to contaminants in winter.

The weak mixing and VOC pollutions dispersion were usual as wind rates were less than 3 m/s at samples period (Figs. 2 and 3). The most considerable level of TVOC was related to WTS and TVOC levels was reduced with increasing distance from the WTS. The mean values within 1600 m downwind of the WTS were up to 93 and 115 μg.m^−3^ in 1st March and 10th July, respectively, that were more than similar articles. At 70 m distance of WTS, IOA is 0.85 and RMSE is 2.16 and they were 0.82 and 2.12, respectively for 630 m distance of WTS that indicates excellent performance of TAPM [19]. Generating natural obstacles and above all planting curtains of trees can be applied to decrease the influence of odours and to limit the VOCs dispersion, this process previously used for landing in Canada [25].

## Conclusions

This research was aimed to determine a model for dispersion and VOCs potential health risks for personnel of WTS site. GC-MS was used to analyze collected gas samples to detect and estimate carcinogenic and non-carcinogenic health risks of VOCs.

Health risks valuation showed that WTS personnel might be affected by both acute and chronic health Risks. HR more than 1 for non-cancer risk confirms adverse chronic effect on personnel health. The LCR values more than 1E-05 suggests possible cancer risks for the nearby personnel and residents. Therefore, several controlling approaches should be used for protecting the personnel and residents against non-cancer and cancer risks. Dispersion of emitting VOCs from WTS was modeled using TAPM V4. The harmful health influence of VOCs on human in summer is more significant comparing to winter as the dominant direction of wind is towards high population density areas in summer.

## Abbreviations

EGM: Eulerian grid module
GC-MS: Gas chromatography-mass spectrometry
HR: Hazard ratio
LCR: The lifetime cancer risk
LOD: Limits of detection
LPM: Lagrangian particle module
MSW: Municipal solidwastes
SOAs: Secondary organic aerosols
TAPM: The air pollution modeling
UR: Unit risk
USEPA: US Environmental Protection Agency
VOC: Volatile organic compounds
WTS: Waste transfer station
